# Biochemical Quality Profile of Black Tea from Upper Assam and North Bank Region of Assam, India

**DOI:** 10.3390/foods15010158

**Published:** 2026-01-03

**Authors:** Podma Pollov Sarmah, Himangshu Deka, Priyanuj Parasar, Rashmi Baruah, Santanu Sabhapondit, Dibyajit Buragohain

**Affiliations:** 1 Biochemistry Department, Tocklai Tea Research Institute, Jorhat 785008, Assam, India; pparasar02@gmail.com (P.P.); santanusabhapondit1@gmail.com (S.S.); 2 College of Sericulture, Assam Agricultural University, Jorhat 785013, Assam, India; rashmi.baruah@aau.ac.in; 3 Advisory Department, Tocklai Tea Research Institute, Jorhat 785008, Assam, India; buragohaind67@gmail.com

**Keywords:** black tea, caffeine, CTC, orthodox, polyphenol, theaflavin, water extract

## Abstract

Black tea quality is fundamentally determined by its biochemical composition, providing essential baseline data for producers, traders, and consumers. This study comprehensively evaluates key biochemical quality components of 32 black tea samples from Upper Assam and North Bank regions of Assam, India, produced by both orthodox and CTC methods, using standardized International Organization for Standardization (ISO) analytical protocols for total polyphenols (TP), theaflavins, catechins, water extract (WE), caffeine, thearubigins (TR), theanine, crude fibre (CF), and ash characteristics. The results reveal substantial variation in TP (83.54–184.52 mg g^−1^, avg. 134.07 mg g^−1^), theaflavins (4.88–15.54 mg g^−1^, avg. 8.61 mg g^−1^), caffeine (15.51–39.24 mg g^−1^, avg. 30.09 mg g^−1^), and theanine (2.47–8.16 mg g^−1^, avg. 5.53 mg g^−1^), demonstrating substantial biochemical variation reflecting differences in cultivation practices, leaf maturity, processing conditions and agroclimatic conditions. The orthodox and CTC methods yielded comparable WE (avg. 404.34 vs. avg. 407.91 mg g^−1^) and theanine levels (avg. 5.65 vs. avg. 5.35 mg g^−1^) indicating that both processing types successfully retain key quality components. All analyzed biochemical attributes with established minimum or maximum limits set by the ISO and Food Safety and Standards Authority of India (FSSAI) demonstrated compliance with national and international quality standards. These findings establish contemporary benchmarks for key quality indicators in Assam black teas and confirm the consistency of quality across diverse processing methodologies and cultivation practices.

## 1. Introduction

Black tea, processed from the leaves of *Camellia sinensis* (L.) *Kuntze,* accounts for most of the global tea consumption due to its organoleptic qualities and health benefits. The classification of tea products primarily relies on processing methodology, giving rise to distinct categories such as green, black, oolong, white, pu-erh, and yellow teas [1]. Black tea dominates the global tea market with approximately 78% market share [2]. The chemical composition critically determines black tea quality, with polyphenolic compounds, particularly theaflavins and thearubigins, along with amino acids and volatile components, forming the key determinants of product characteristics [3]. Crude fibre (CF) and water-extractable solids represent additional parameters that influence final product quality [4,5].

Assam stands as India’s largest tea-producing state, accounting for over half of the national black tea output. The region produced more than 649 million kg of processed tea in 2024 [6]. Domestically, approximately 80% of Assam’s tea production serves local markets [7]. Both orthodox and CTC (Crush, Tear and Curl) manufacturing methods are employed in Assam, producing teas with distinct sensory and chemical profiles. Geographic variation within Assam itself is pronounced, with Upper Assam, North Bank, South Bank, and Barak Valley representing distinct production zones characterized by different soil types, elevations, and microclimates [7]. These terrain specific variations create recognizable quality signatures across regions, influencing the phenolic profiles and oxidation potential of harvested leaves.

The tea leaf composition undergoes significant biochemical modifications during black tea processing. Polyphenolic transformation represents the most notable change. Fresh leaves contain catechins as major polyphenolic constituents, representing 70–80% of total polyphenol (TP) content. The principal catechin variants include epigallocatechin gallate (EGCG), epicatechin gallate (ECG), epigallocatechin (EGC), epicatechin (EC), and (+)-catechin (+C) [8]. The leaf processing induces enzymatic oxidation of these catechins through the action of polyphenol oxidase (PPO) and peroxidase (PO), converting them into theaflavins and thearubigins. These resulting compounds define sensory attributes like astringency, briskness, strength, etc., and substantially influence consumer perception and product value. The extent and composition of this transformation depend on the initial leaf biochemistry and manufacturing conditions applied.

Across Assam’s regions, young tea shoots display considerable variation in biochemical constituents. Cultivar selection, local temperature fluctuations, precipitation patterns, solar radiation, nutrient availability, and harvest timing collectively shape the chemical profile of harvested leaves [9]. Leaf age represents a particularly significant factor. Amino acids accumulate preferentially in young shoot and decline as leaves mature [10]. Catechin concentrations similarly fluctuate based on leaf maturity and cultivar.

Prior research has established substantial quality variation across Assam’s tea-producing regions. Bhuyan et al. documented polyphenolic variation in teas from the Brahmaputra and Barak valleys [9]. Bhuyan et al. subsequently provided a comparative analysis of Assam’s major regions, Upper Assam, North Bank, South Bank, and Barak Valley, characterizing their respective quality profiles [7]. Deka et al. more recently examined quality parameters across different cultivars and leaf maturity stages, identifying pronounced differences in theaflavin, thearubigin, catechin, caffeine, and theanine levels [3,11].

Despite Assam’s commercial significance as an Indian tea-producing state, current data on quality metrics from its principal regions remains notably scarce. The most recent large-scale assessment occurred over a decade ago [7]. Since then, agricultural methodologies, processing technologies, and environmental conditions have evolved. Current quality benchmarking against International Organization for Standardization (ISO) and Food Safety and Standards Authority of India (FSSAI) standards presents a significant information gap. Producers require contemporary data to refine cultivation strategies and manufacturing approaches. Traders and exporters benefit from the updated information for market positioning and international competitiveness. Consumers increasingly seek knowledge of nutritional composition and sensory properties across regional origins. Systematic, current evaluation of Assam’s tea quality thus serves multiple stakeholder interests.

The Upper Assam and North Bank regions represent particularly important areas, both economically and in terms of production volume, yet their recent quality profiles remain inadequately documented. Addressing this knowledge gap through systematic characterization of key biochemical constituents in black tea from these zones, incorporating both orthodox and CTC processing methods, provides essential baseline data. To address the scarcity of recent data, this study aims to update the biochemical quality parameters of orthodox and CTC black teas, and assesses conformance to international quality standards.

## 2. Materials and Methods

### 2.1. Chemicals

HPLC-grade standard such as (−)-epigallocatechin (≥95%), (+)-catechin (≥95%), (−)-epicatechin (≥90%), (−)-epigallocatechin-3-gallate (≥95%), (−)-epicatechin-3-gallate (≥95%), caffeine (anhydrous, 99%), gallic acid monohydrate (≥98.0%), and L-theanine were purchased from Sigma-Aldrich, Steinheim, Germany. Folin–Ciocalteu Phenol (FCP) reagent (LR) was procured from HiMedia Laboratories, India. Acetic acid (HPLC grade), acetonitrile (HPLC grade), methanol (HPLC grade), sodium carbonate (AR) were purchased from Merck KGaA, Darmstadt, Germany. Sulphuric acid (AR), hydrochloric acid (AR), oxalic acid (AR), Na_2_HPO_4_ (AR) and all other chemicals of AR grade were purchased from SRL, Mumbai, India. HPLC-grade water from SRL, Mumbai, India was used for the preparation of HPLC eluents and extraction solution of various analysis.

### 2.2. Instrumentation

A HPLC system (Make: ThermoFisher Scientific, Germering, Germany; Model: Vanquish Core) was used for analysis and quantification of caffeine, catechin, theaflavin, and theanine in the samples. The HPLC system was fitted with a Phenomenex Luna 5 μ phenylhexyl column (4.5 mm × 250 mm) along with a UV-Vis detector and autosampler unit. For theanine analysis, a Acclaim^TM^ 120, C18 5 μ (3.0 mm × 150 mm) column (Make: ThermoFisher Scientific, Vilnius, Lithuania) was used. The column temperature was maintained at 25 ± 0.5 °C and detector wavelengths were set at 210 nm for theanine and 278 nm for both catechin and caffeine analysis. Spectroscopic analyses were carried out using a spectrophotometer (Make: Varian, New South Wales, Australia; Model: Cary 50). Water from Millipore Milli-Q Synthesis (Make: Merck, Darmstadt, Germany) was used for various experimental work.

### 2.3. Tea Sampling and Sample Preparation for Analysis

**Figure 1:**
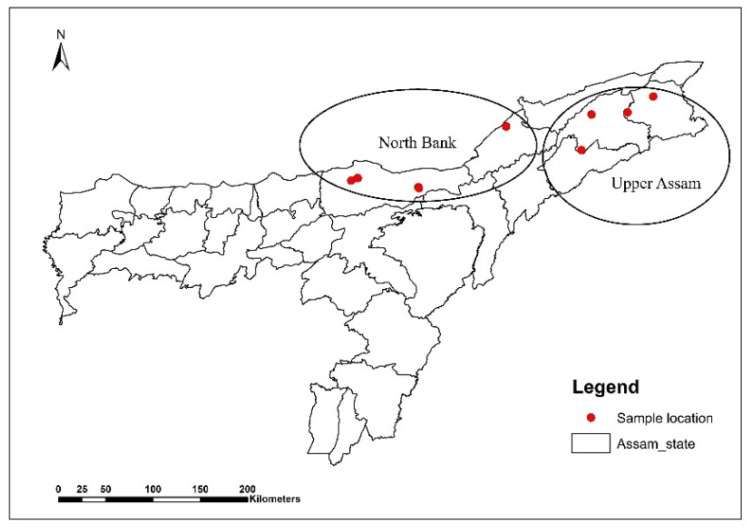
Sampling locations from Upper Assam and North Bank region of Assam, India.

Freshly processed black tea samples, including both orthodox and CTC types (32 in total), were collected from commercial tea estates in two distinct agroclimatic regions of Assam, India—Upper Assam and North Bank—during April–May, 2023. Samples were vacuum-sealed in aluminum foil packs to protect against light and moisture exposure during transportation and storage. Samples from the Upper Assam region were coded U1 to U20 (20 samples), while those from the North Bank region were coded N1 to N12 (12 samples). Figure 1 illustrates the sampling regions and the locations of the respective estates. Weather data for the sampling period in both regions are provided in the Supplementary Information (Table S1). Immediately after collection, all samples were carried to the laboratory and stored at −20 °C until further analysis. The samples were kept at room temperature under sealed conditions for 24 h before analysis. The tea samples were grinded in a grinding mill fitted with sieve of 500 µm. The powdered samples passing through the sieve were collected into an airtight container and used for analysis. The moisture content was measured in the sample as per the procedure laid out in Section 2.4. All analytical data presented in the manuscript are expressed on a dry matter basis and were corrected using a moisture correction factor derived from moisture analysis.

### 2.4. Determination of Dry Matter Content

An empty weighing bottle fitted with a lid was heated at 103 ± 2 °C in a hot air oven for 1 h, cooled in a desiccator, and weighed using an analytical balance. This procedure was repeated until a constant weight was obtained. Approximately 5 g of powdered tea sample, weighed to the nearest 0.001 g, was transferred into the calibrated weighing bottle and dried at 103 ± 2 °C in a hot air oven for 4 h. After drying, the bottle was cooled in a desiccator and weighed. The drying and weighing steps were repeated until a constant weight was achieved. Moisture content was calculated from the difference in weights before and after drying.

### 2.5. Determination of Total Polyphenol Content

Determination of TP content in tea samples was accomplished following the ISO methodology [12]. In brief, 0.20 g finely ground samples underwent sequential extraction employing 5 mL aliquots of 70% methanol solution at 70 °C in a water bath fitted with thermostat. The extracted volume was brought to 10 mL through addition of extraction medium in volumetric flask. The prepared extract, after 100-fold dilution, was then combined with 5 mL of FCP reagent (10% *v*/*v* in water). After 3–4 min, 4 mL of Na_2_CO_3_ solution (7.5% *w*/*v*) was added into the mixture and agitated to ensure uniform mixing. The prepared reaction system was maintained at ambient temperature for a duration of 1 h followed by measuring absorbance at 765 nm using spectrophotometer. For reference, a blank control was prepared by substituting the tea extract with distilled water. The TP content quantification was performed by comparison with a gallic acid reference standard curve (y = 0.012x + 0.0121, R^2^ = 0.9999).

### 2.6. Determination of Catechin and Caffeine Content

Caffeine and catechin concentrations in the tea samples were quantified following the ISO reference procedure [13]. Briefly, 0.20 g of finely powdered tea was extracted twice using 5 mL of aqueous methanolic solution (70%, *v*/*v*). The extracted volume was brought to 10 mL through addition of extraction medium in volumetric flask. Prior to chromatographic analysis, the extracts were diluted five times followed by filtration through a 0.20 μm membrane filter. Quantitative determination of caffeine and individual catechins was carried out by HPLC. Separation was achieved using a binary mobile phase system comprising solvent A (water containing 9% acetonitrile and 2% acetic acid) and solvent B (80% acetonitrile with 2% acetic acid). The elution was performed at a flow rate of 1 mL min^−1^, starting with isocratic conditions of 100% solvent A for 10 min, followed by a linear gradient to 68% solvent A and 32% solvent B over 15 min, and maintained at this composition for further 10 min. Identification of caffeine and catechin peaks was accomplished by comparison with reference standards, and quantification was performed using relative response factors with caffeine as specified in the ISO method.

### 2.7. Determination of Theanine Content

The concentration of theanine in tea samples was measured in accordance with the ISO protocol [14]. Briefly, 1.0 g of finely powdered tea was extracted with 100 mL of boiling distilled water under continuous stirring for 5 min. The extract was subsequently filtered, and the resulting solution was passed through a 0.20 μm membrane filter prior to chromatographic analysis. An aliquot of 20 μL of the filtered extract was injected into the HPLC system. Separation was carried out using water as the sole mobile phase with an elution time of 22 min followed by column cleanup using 80% acetonitrile for 10 min. Equilibration with water was performed for 13 min before next analysis. Quantification of theanine was achieved by external calibration, using a standard curve constructed from theanine solutions in the concentration range of 10–100 μg mL^−1^ (y = 0.1131x + 0.4561, R^2^ = 0.999).

### 2.8. Determination of Water Extract

The water extract (WE) content of black tea samples was determined using previously reported procedure, with slight modifications [15]. In brief, 2.0 g of tea samples were refluxed with hot water for 1 h with intermittent stirring, followed by filtration through a pre-weighed sintered crucible. The residue was subsequently washed with 200 mL of hot water, and the crucible containing the residue was dried in a hot air oven at 103 ± 2 °C for 16 h, cooled in a desiccator, and weighed. This procedure was repeated with 1 h heating until a constant weight was obtained. The WE (%) was calculated based on the difference in mass between the initial tea sample and the water-insoluble residue.

### 2.9. Determination of Crude Fibre

The CF content of the tea samples was determined according to the ISO standard [16]. 2.0 g of finely ground tea samples were placed in a conical flask with 200 mL of 1.25% (*w*/*v*) sulfuric acid, then boiled for 30 min under reflux on a hot plate. The residue was filtered through a Whatman filter paper, thoroughly washed with water, and returned to the flask, and the process was repeated using 1.25% (*w*/*v*) sodium hydroxide in place of acid. The insoluble residue was transferred to a pre-weighed sintered crucible (porosity No. 1, P160) with boiling water, washed sequentially with ethanol and acetone, and dried at 103 ± 2 °C in a hot-air oven to constant weight as described in Section 2.8. CF content was calculated from the difference in weight.

### 2.10. Determination of Thearubigin Content

The thearubigin (TR) content in black tea samples was determined using the method reported by Ullah [17]. Briefly, a tea infusion was prepared by steeping 6.0 g of tea in 250 mL boiling water in a thermoflask for 10 min, followed by filtration and cooling. A 6 mL aliquot of infusion was combined with an equal volume of 1% (*w*/*v*) Na_2_HPO_4_ solution, then 10 mL ethyl acetate was added and vigorously shaken for 1 min, with an additional 5 mL ethyl acetate added. Next, 10 mL of the ethyl acetate layer was diluted to 25 mL with methanol in a volumetric flask (E_1_). Separately, 1 mL infusion, 1 mL of 10% (*w*/*v*) oxalic acid, and 8 mL water were mixed and adjusted to 25 mL with methanol (E_2_). Absorbances of E_1_ and E_2_ were recorded at 380 nm. TR content was calculated using the following formula.

$$Thearubigin\;content\left(\%\right)=7.06\times (4E_{2}-E_{1})$$

### 2.11. Determination of Theaflavin Profile

Individual theaflavins in tea samples were quantified by HPLC according to the ISO procedure [18]. 0.20 g of finely ground tea samples underwent double extraction with 5 mL portions of 70% methanol, and the combined extract was adjusted to 10 mL in a volumetric flask. This extract received five-fold dilution followed by filtration through a 0.20 μm membrane filter before injection. Separation employed solvent A (1% acetic acid in acetonitrile) and solvent B (1% acetic acid in water) in isocratic mode (75:25, *v*/*v*) for 30 min. Peaks were identified via reference standards, with quantification based on a caffeine calibration curve (y = 0.3126x + 0.2767, R^2^ = 0.9995) and method-specified relative response factors.

### 2.12. Determination of Total Ash and Its Profile

#### 2.12.1. Determination of Total Ash

Five gram (5.0 g) of finely ground tea sample was weighed accurately (±0.001 g) into a previously ignited, cooled, and weighed dish [19]. The sample was first dried near 100 °C to remove moisture, then incinerated in a muffle furnace at 525 ± 25 °C until the ash appeared free from carbon (usually after about 2 h). The residue was cooled in a desiccator, moistened with distilled water, dried on a hot plate, and reheated in the furnace for 1 h. This process of heating, cooling, and weighing was repeated until two successive weights differed by no more than 0.001 g. The total ash was reserved for further determinations of water-soluble, water-insoluble, or acid-insoluble ash. The total ash content, expressed as a percentage of dry matter, was calculated using the formula:

$$\mathrm{Total}\;\mathrm{ash}\;(\mathrm{in}\;\%)\;=\;\frac{m_{1}\times 100\times 100}{m_{0}\times DM}$$

where m_0_ is the mass of the test portion (in g), m_1_ is the mass of total ash (in g), and DM is the dry matter content (in %).

#### 2.12.2. Determination of Water-Insoluble and Water-Soluble Ash

The total ash obtained as per the previous Section 2.12.1 was treated with 20 mL of distilled water and heated nearly to boiling. The mixture was filtered through ash-free filter paper, and the dish and residue were thoroughly washed with hot distilled water until the combined filtrate and washings measured about 60 mL. The filter paper with its contents was returned to the dish, evaporated to dryness on a steam bath, and ignited in a muffle furnace at 525 ± 25 °C until free from carbon. The residue was cooled in a desiccator, weighed, reheated for 30 min, and reweighed until a constant weight was obtained. The filtrate was reserved for the determination of the alkalinity of the water-soluble ash, and the residue for the determination of acid-insoluble ash [20].

$$\mathrm{Water}-\mathrm{insoluble}\;\mathrm{ash}\;(\mathrm{in}\;\%)=\frac{m_{2}\times 100\times 100}{m_{0}\times DM}$$

where m_0_ is the mass of the test portion (in g), m_2_ is the mass in grams of the water-insoluble ash (in g), and DM is the dry matter content (in %).

The water-soluble ash was calculated using the formula

$$\mathrm{Water}-\mathrm{soluble}\;\mathrm{ash}\;(\mathrm{in}\;\%)=\frac{\left(m_{1}-m_{2}\right)\times 100\times 100}{m_{0}\times DM}$$

where m_0_ is the mass of the test portion (in g), m_1_ is the mass of total ash (in g), m_2_ is the mass of the water-insoluble ash (in g), and DM is the dry matter content (in %).

#### 2.12.3. Determination of Acid-Insoluble Ash

The total ash obtained as described in Section 2.12.1 was treated with 25 mL of hydrochloric acid solution in a silica dish, covered with a watch glass, and gently boiled for 10 min. After cooling, the contents were filtered through ash-free filter paper, and both the dish and residue were washed thoroughly with hot distilled water until the washings were free from acid (confirmed using silver nitrate solution). The filter paper with its contents was returned to the dish, evaporated to dryness on a boiling water bath, and ignited in a muffle furnace at 525 ± 25 °C until free from carbon. The dish was cooled in a desiccator and weighed. The heating, cooling, and weighing steps were repeated until a constant weight was obtained [21]. The acid-insoluble ash content, expressed as a percentage of dry matter, was calculated using the formula:

$$\mathrm{Acid}-\mathrm{insoluble}\;\mathrm{ash}\;(\mathrm{in}\;\%)\;=\;\frac{m_{3}\times 100\times 100}{m_{0}\times DM}$$

where m_0_ is the mass of the test portion (in g), m_3_ is the mass of the acid-insoluble ash (in g), and DM is the dry matter content (in %).

#### 2.12.4. Determination of Alkalinity of Water-Soluble Ash

The filtrate obtained from the determination of water-soluble ash as in Section 2.12.2 was cooled and titrated with 0.1 N hydrochloric acid solution using methyl orange as the indicator. Two parallel determinations were carried out using filtrates from duplicate ash determinations to ensure accuracy and repeatability [22].

The alkalinity of the water-soluble ash was expressed in terms of milliequivalents per 100 g of dry sample or as a percentage by mass of potassium hydroxide (KOH) on a dry basis. It was calculated using the formula

$$\mathrm{Alkalinity}\;\mathrm{of}\;\mathrm{the}\;\mathrm{water}–\mathrm{soluble}\;\mathrm{ash}=\;\frac{V\times 100\times 100}{10\times m_{0}\times DM}$$

where V is the volume (in mL) of 0.1 N HCl used in titration, m_0_ is the mass (in g) of the sample used for total ash determination, and DM is the dry matter content (in %).

### 2.13. Statistical Analysis

All results are presented as the ‘mean ± standard deviation’ (SD) of three independent measurements. The WASP–Web Agri Stat Package 2.0 (Developed at ICAR Research Complex for Goa, Goa, India) was used to compare mean values of the measured parameters across samples. Differences among the biochemical parameters of individual samples were assessed using Tukey’s multiple comparison test at significance levels of *p* ≤ 0.01 and *p* ≤ 0.05. Pearson correlation heatmaps among biochemical constituents and sieve mesh size (orthodox teas) were generated using Origin (Version 2024b). Box plot analyses of biochemical data were performed in SPSS Version 18.0 (SPSS Inc., Chicago, IL, USA). 

## 3. Results and Discussion

The biochemical composition of black tea serves as the fundamental determinant of its quality and market value. This study comprehensively evaluated thirty-two black tea samples from the Upper Assam and North Bank regions of Assam, India to characterize key quality attributes like polyphenols, theaflavins, caffeine, and water extract, etc. The analysis revealed distinct variations in these biochemical constituents, reflecting the influence of regional agro-climatic conditions and processing methodologies. The analytical data for these biochemical parameters are presented in Table 1 and Table 2.

### 3.1. Total Polyphenol

**Figure 2:**
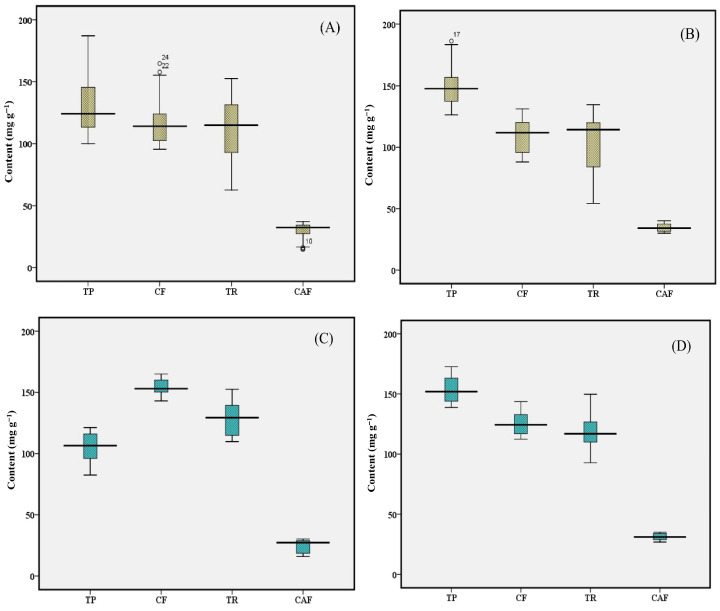
Box-plot diagrams showing the mean values and ranges of TP, CF, TR, and caffeine in orthodox black tea: (**A**) Upper Assam and (**B**) North Bank; and in CTC black tea: (**C**) Upper Assam and (**D**) North Bank. TP, total polyphenol; CF, crude fibre; TR, thearubigins; CAF, Caffeine.

Polyphenols in tea are well known for their health-beneficial effects, particularly for antioxidant activity. Among the different types of polyphenols, catechins play a pivotal role in promoting human health. EGCG has gained special attention due to its dominant presence and anti-cancer, anti-inflammatory properties, etc. The TP content in orthodox black tea samples from the Upper Assam and North Bank region ranged from 100.29 to 184.52 mg g^−1^, with an average of 136.89 mg g^−1^, indicating a 1.84-fold variation, and the data are presented in Figure 2. The Upper Assam region and North bank region showed average TP content of 131.15 and 150.29 mg g^−1^, respectively. These TP levels were well above the minimum requirement of 9% (equivalent to 90.0 mg g^−1^) set by ISO standards [5]. The findings are consistent with Deka et al., who observed TP levels in black tea processed from eight different cultivars in the range from 133.6 to 167.6 mg g^−1^ while demonstrating the changes during processing of tea [11]. During orthodox tea processing, catechins undergo oxidation and polymerization to form theaflavins and thearubigins, though the extent of catechin reduction is less intensive than other processing methods. The total catechin (TC) content in orthodox black tea samples varied considerably. Individual catechins EGCG, ECG, EGC, EC and +C were detected across a broad range, with EGCG ranging from 0.86 ± 0.10 to 12.34 ± 0.39 mg g^−1^, ECG from 2.10 ± 0.13 to 13.35 ± 0.38 mg g^−1^, EGC from below the detectable limit (BDL) to 2.98 ± 0.19 mg g^−1^, EC from BDL to 7.40 ± 0.66 mg g^−1^, and +C from BDL to 0.67 ± 0.04 mg g^−1^ [Table S2 (Supplementary Information File)]. The variation in polyphenolic content in orthodox teas depends on agroclimatic conditions of the region where the tea plant is cultivated, including factors such as temperature, sunlight, and rainfall [23].

The TP content in CTC black tea samples from the regions ranged from 83.54 to 169.74 mg g^−1^, with an average of 129.37 mg g^−1^, representing a 2.03-fold variation across the twelve samples analyzed (Figure 2C,D). The average TP contents in CTC black tea samples from the Upper Assam and North Bank regions were 104.32 and 154.41 mg g^−1^, respectively. All TP levels exceeded the minimum requirement of 9% (equivalent to 90.0 mg g^−1^) set by ISO standards [5]. These findings align with Deka et al. who reported TP content in CTC black tea processed from leaves of five different cultivars with varying maturity ranging from 104.96 to 167.83 mg g^−1^ [3]. Bhuyan et al. also reported TP levels in black tea originating from Brahmaputra valley (86.2 to 151.8 mg g^−1^) consistent with the current observations for CTC black teas [9]. During CTC processing, catechins are oxidized and polymerized to form theaflavins and thearubigins, with catechin levels decreasing to an extent of 96% [3]. Consequently, the level of catechins in CTC black tea samples remains very low. In CTC samples, TC content varied from BDL to lower concentration ranges. The substantial reduction in catechin levels reflects the intensive oxidation characteristic of CTC manufacturing, which promotes the formation of theaflavins and thearubigins, compounds responsible for the color, astringency, and antioxidant properties of black tea.

### 3.2. Water Extract

WE in tea is the amount of biochemicals that are extracted in hot water during brewing and is a critical indicator of infusion quality. It provides depth to the infusion and reflects the overall quality of the tea. The WE level across all black tea samples ranged from 349.29 ± 9.07 to 447.54 ± 9.85 mg g^−1^, with an average of 405.68 mg g^−1^, representing a 1.28-fold variation (Table 1). This variation reflects differences in leaf biochemistry, processing methodology, and brewing characteristics across the samples analyzed. The average WE content of orthodox and CTC tea was almost similar, with values of 404.34 and 407.91 mg g^−1^, respectively. The WE level of all the tea samples was higher than the minimum limit of 32% (equivalent to 320.00 mg g^−1^) set by the FSSAI [24] and ISO [5], indicating compliance with national and international quality. The consistency of WE content across diverse samples demonstrates the stability and quality of the tea samples analyzed.

### 3.3. Caffeine

Caffeine is a key alkaloid in tea that forms complexes with theaflavins through hydrogen bonding, which imparts a fresh and brisk taste to the beverage [25]. The biosynthesis of caffeine primarily takes place in young leaves, with its content decreasing with the maturity of leaves [3]. Across all black tea samples analyzed, caffeine content ranged from 15.51 ± 1.07 to 39.24 ± 1.49 mg g^−1^, with an average of 30.09 mg g^−1^. Caffeine content in black teas from the Upper Assam region ranged from 15.51 ± 1.07 to 36.88 ± 0.41 mg g^−1^, with an average of 28.40 mg g^−1^, representing a 2.38-fold variation (Figure 2). In the North Bank region, caffeine levels were relatively higher, ranging from 28.10 ± 0.54 to 39.24 ± 1.49 mg g^−1^, with an average of 32.91 mg g^−1^, and a 1.40-fold variation. The average caffeine contents of orthodox and CTC teas were 29.84 and 25.05 mg g^−1^ in Upper Assam, and 34.58 and 31.24 mg g^−1^ in North Bank, respectively. These current findings of caffeine levels are consistent with earlier studies of black tea processed from different cultivars (35–55 mg g^−1^) and from leaves of varying maturity and cultivars (24–54 mg g^−1^) [3,11]. Moreover, in another study of Congou black tea, caffeine levels ranged from 3.75 to 4.50% (equivalent to 37.5–45.0 mg g^−1^), which falls within the range observed in the current study. The substantial variation observed in the present study is thus due to differences in leaf maturity at harvest, plucking standards, and cultivation practices, which ultimately influence caffeine content in the processed product.

### 3.4. Crude Fibre

The CF in tea originates from structural components of plants such as cell walls, vascular tissues, collenchyma, and sclerenchyma, and constitutes an important aspect of tea quality and composition. CF is affected by multiple factors including leaf maturity, plucking interval, and processing method [3,9] and collectively determine the CF content in the processed tea. Across all black tea samples, the CF content ranged from 91.87 ± 3.84 to 162.71 ± 2.08 mg g^−1^, with an average of 125.17 mg g^−1^, representing a 1.77-fold variation. In Upper Assam, CF levels ranged from 97.33 ± 1.90 to 162.71 ± 2.08 mg g^−1^, while in the North Bank region, it ranged from 91.87 ± 3.84 to 139.19 ± 4.49 mg g^−1^ (Figure 2). The average CF contents of orthodox and CTC teas were 117.79 and 154.33 mg g^−1^, respectively, in Upper Assam, and 109.20 and 125.56 mg g^−1^, respectively, in the North Bank. All samples in the current study fell within the permissible limit of 16.5% (165 mg g^−1^) specified by the FSSAI [24] and ISO standards for black tea [5], indicating compliance with both national and international specifications. Similarly, Kc et al. reported comparable CF levels (88.2–154.7 mg g^−1^) in black teas from Nepal [26], which are consistent with the present findings.

### 3.5. Thearubigin

TRs are formed through polymerisation of theaflavins and catechins during the processing of black tea [1]. The formation of TR is directly influenced by the processing method, particularly the fermentation stage of tea manufacture and the degree of oxidation achieved. TR is a critical quality-defining compound that contributes significantly to the color, strength, and mouthfeel of the tea infusion, making it an important determinant of tea sensory attributes [9,25]. Across all black tea samples, TR levels ranged from 59.62 ± 4.73 to 145.37 ± 6.22 mg g^−1^, with an average of 114.49 mg g^−1^, showing a 2.44-fold variation. TR levels in tea samples from the Upper Assam region ranged from 73.95 ± 12.14 to 145.37 ± 6.22 mg g^−1^, with an average of 116.84 mg g^−1^ and in North Bank region, TR levels ranged from 59.62 ± 4.73 to 137.84 ± 11.86 mg g^−1^, with an average of 110.58 mg g^−1^ (Figure 2).

The present findings align with those of Deka et al., who reported TR content in black tea processed from different cultivars ranging from 71.57 to 148.90 mg g^−1^, which is similar to the range observed in the current study [3]. However, a previous study by Bhuyan et al. observed higher TR levels (105.6 to 183.5 mg g^−1^) for black tea samples [9]. In contrast, Congou black tea processed with varying rolling durations showed substantially lower TR levels of 30.48 to 51.96 mg g^−1^, highlighting the significant influence of processing techniques as well as agroclimatic conditions on TR formation [27].

### 3.6. Theanine

**Figure 3:**
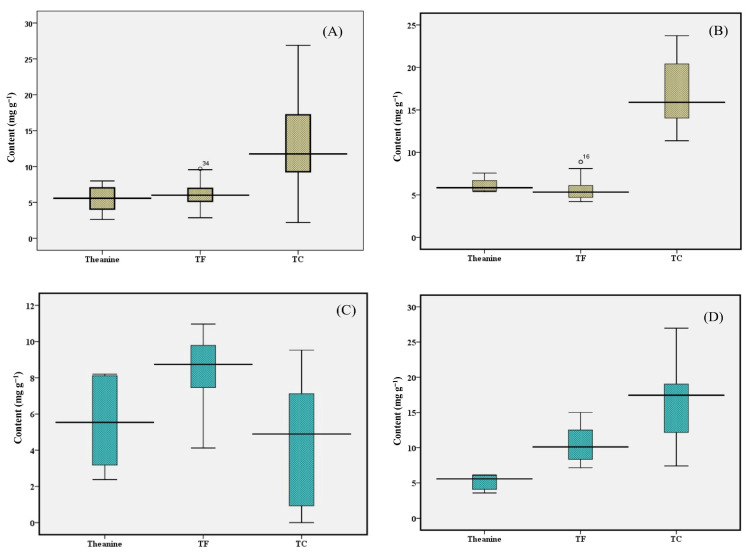
Box-plot diagrams showing the mean values and ranges of Theanine, TF, and TC in orthodox black tea from (**A**) Upper Assam and (**B**) North Bank, and in CTC black tea from (**C**) Upper Assam and (**D**) North Bank. TF, total theaflavin; TC, total catechin.

Theanine is a unique amino acid predominantly found in tea leaves and plays a critical role from both nutritional and sensory perspectives. It significantly contributes to the sensory profile of tea and also possesses several health benefits. Factors such as cultivation practice, light intensity, soil nutrition, and particularly ammonia levels, environment, and growing season significantly influence the biosynthesis of the amino acid in tea plants [3,28]. Theanine alone contributes around 50% to total amino acid content in tea leaves. In the present study, a wide variation was observed in the theanine content, ranging from 2.47 ± 0.08 to 8.16 ± 0.03 mg g^−1^, with an average of 5.53 mg g^−1^ and a 3.30-fold variation (Figure 3). The average theanine content in orthodox teas showed almost similar content to that of CTC tea, with a value of 5.65 and 5.35 mg g^−1^, respectively. The theanine content of Upper Assam teas, ranging from 2.47 ± 0.08 to 8.16 ± 0.03 mg g^−1^, with an average of 5.45 mg g^−1^ and a 3.30-fold variation, indicates considerable influence of cultivation and processing practices. The average theanine contents of orthodox and CTC teas of the Upper Assam region were 5.42 and 5.52 mg g^−1^ whereas the North Bank region showed 6.16 and 5.18 mg g^−1^, respectively.

### 3.7. Theaflavin Profile

**Figure 4:**
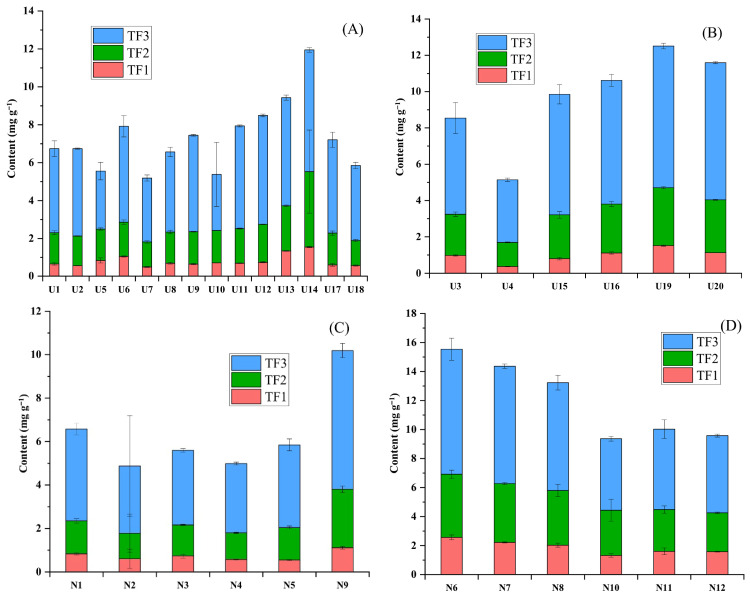
TF profiles of orthodox and CTC black tea from Upper Assam and the North Bank region: (**A**) Orthodox, Upper Assam, (**B**) CTC, Upper Assam, (**C**) Orthodox, North Bank, and (**D**) CTC, North Bank. TF1, theaflavin; TF2, theaflavin monogallate; TF3, theaflavin digallate.

Theaflavin is a critical quality-defining parameter for black tea, primarily responsible for its astringency, briskness, and bright orange-yellow color of liquor [29]. Theaflavins are formed through a PPO-catalyzed oxidative polymerisation reaction of dihydroxylated and trihydroxylated catechins, during the processing of black tea. The content of individual theaflavins, namely theaflavin (TF1), theaflavin monogallate (TF2), and theaflavin digallate (TF3), was quantified in all black tea samples. The sum of these contents was expressed as the total theaflavin (TF) content. The theaflavin profile of the tea samples is presented in Figure 4. Across all samples analyzed, TF1 content ranged from 0.37 ± 0.02 to 2.57 ± 0.17 mg g^−1^. TF2 ranged from 1.17 ± 0.87 to 4.35 ± 0.28 mg g^−1^, while TF3 varied between 2.96 ± 0.69 and 8.62 ± 0.76 mg g^−1^. The TF content across all samples ranged from 4.88 ± 0.64 to 15.54 ± 1.20 mg g^−1^, with an average of 8.61 mg g^−1^ (Figure 3). In samples from the Upper Assam region, TF1 content ranged from 0.37 ± 0.02 to 1.55 ± 0.03 mg g^−1^, with an average of 0.85 mg g^−1^. TF2 ranged from 1.32 ± 0.03 to 3.98 ± 0.19 mg g^−1^ (average 2.06 mg g^−1^), while TF3 varied between 2.96 ± 0.69 and 7.80 ± 0.16 mg g^−1^ (average 5.13 mg g^−1^). The TF content in this region ranged from 5.14 ± 0.11 to 12.51 ± 0.25 mg g^−1^, with an average of 8.03 mg g^−1^. The tea samples from the North Bank region exhibited TF1 content ranged from 0.55 ± 0.03 to 2.57 ± 0.17 mg g^−1^ (average 1.31 mg g^−1^), TF2 from 1.17 ± 0.87 to 4.35 ± 0.28 mg g^−1^ (average 2.53 mg g^−1^), and TF3 from 3.10 ± 0.31 to 8.62 ± 0.76 mg g^−1^ (average 5.34 mg g^−1^). The TF content in the North Bank samples varied from 4.88 ± 0.64 to 15.54 ± 1.20 mg g^−1^, with an average of 9.18 mg g^−1^.

In our earlier study of black tea in five cultivars with varying leaf maturity from two leaves and bud to three leaves and bud, the TF content ranged from 9.91 to 24.10 mg g^−1^, with teas produced from two leaves and bud producing more than 20 mg g^−1^ TF irrespective of cultivars [3], which were much higher than the current findings. However, the present observation of TF is in line with those in Congou black tea (7.0–8.0 mg g^−1^), as observed by Hua et al. [30]. On the other hand, Chiang et al. reported a lower level of TF (3.7–6.8 mg g^−1^) in black tea during an enzyme treatment study [31]. In another study of six Congou black tea samples, Yue et al. reported TF levels in the range from 6.37 to 21.72 mg g^−1^ [4]. Overall, teas from the North Bank region showed relatively higher TF levels compared to those from Upper Assam, indicating a greater extent of oxidative polymerisation during fermentation. This variation may be attributed to regional differences in leaf biochemistry, processing conditions, or environmental factors such as altitude and temperature, which influence enzymatic oxidation and the formation of theaflavins.

### 3.8. Ash Profile

The ash content in tea reflects its mineral composition and is an important indicator of quality, particularly the proportion of water-soluble ash, which contains beneficial minerals and is associated with superior quality. The ash content can vary depending on factors like the tea plant’s growing conditions, processing method, shooting periods, storage conditions, etc. [32].

Across all black tea samples, the total ash content ranged from 5.53 ± 0.21 to 8.42 ± 0.45%, with water-soluble and water-insoluble ash varying between 3.20 ± 0.28 and 4.55 ± 0.20%; and 1.70 ± 0.06 and 3.97 ± 0.31%, respectively (Table 2). The acid-insoluble ash content ranged from 0.02 ± 0.01 to 1.98 ± 0.35% with an average of 0.32%, and the alkalinity of the water-soluble ash varied between 1.80 ± 0.02 and 2.37 ± 0.08 g KOH equivalent. The proportion of water-soluble ash relative to total ash demonstrated good mineral solubility across samples, with values ranging from 45.15 to 71.31%, surpassing the ISO minimum requirement of 45% in all samples [5]. In black tea samples from the Upper Assam region, the total ash content ranged from 5.53 ± 0.21 to 8.42 ± 0.45%, with water-soluble and water-insoluble ash ranging from 3.20 ± 0.28 to 4.55 ± 0.20% and 1.78 ± 0.01 to 3.97 ± 0.31%, respectively. The acid-insoluble ash content ranged from 0.02 ± 0.01 to 1.98 ± 0.35% with an average content of 0.47%, and the alkalinity of the water-soluble ash was between 1.80 ± 0.02 and 2.37 ± 0.08 g KOH equivalent. In tea samples from the North Bank region, the total ash content varied from 5.59 ± 0.21 to 6.34 ± 0.14%, demonstrating lower variability compared to the Upper Assam. The water-soluble and water-insoluble ash fractions ranged from 3.78 ± 0.27 to 4.18 ± 0.17% and 1.70 ± 0.06 to 2.25 ± 0.02%, respectively. The acid-insoluble ash content was comparatively lower (0.04 ± 0.01–0.23 ± 0.03%) with an average content of 0.11%, while the alkalinity of the water-soluble ash ranged from 1.89 ± 0.05 to 2.25 ± 0.03 g KOH equivalent.

The total ash content of tea samples from both regions largely conformed to the ISO specification for black tea (4.0–8.0%) [5], with all North Bank samples and most Upper Assam samples meeting the standard. The proportion of water-soluble ash relative to total ash surpassed the ISO minimum requirement of 45% in all samples, indicating good mineral solubility and quality. Specifically, values ranged from 45.15 to 71.31% in Upper Assam and from 61.30 to 70.32% in the North Bank region. The average acid-insoluble ash content for both regions was within the ISO limit of 1% [5]. Variation in soluble and insoluble ash among the black tea samples can be attributed to differences in the mineral nutrition of tea bushes and estate-specific agronomic and processing practices, which influence the accumulation and retention of inorganic constituents in the harvested shoots and final made tea. Reported work on black teas similarly shows that ash characteristics and mineral composition differ between regions and factories, highlighting the combined impact of soil conditions, fertilization, and manufacturing steps on total and fractional ash values [33].

In contrast, the alkalinity of water-soluble ash was within the ISO-specified range (1–3% as KOH) across all samples, reflecting compliance with acceptable quality parameters [5]. Overall, the Upper Assam teas exhibited higher variability and generally greater total ash and acid-insoluble ash contents compared to the North Bank samples. These differences may reflect regional variations in soil mineral composition, leaf maturity, and manufacturing conditions, which can influence the accumulation of inorganic constituents in tea leaves.

### 3.9. Correlation Analysis

**Figure 5:**
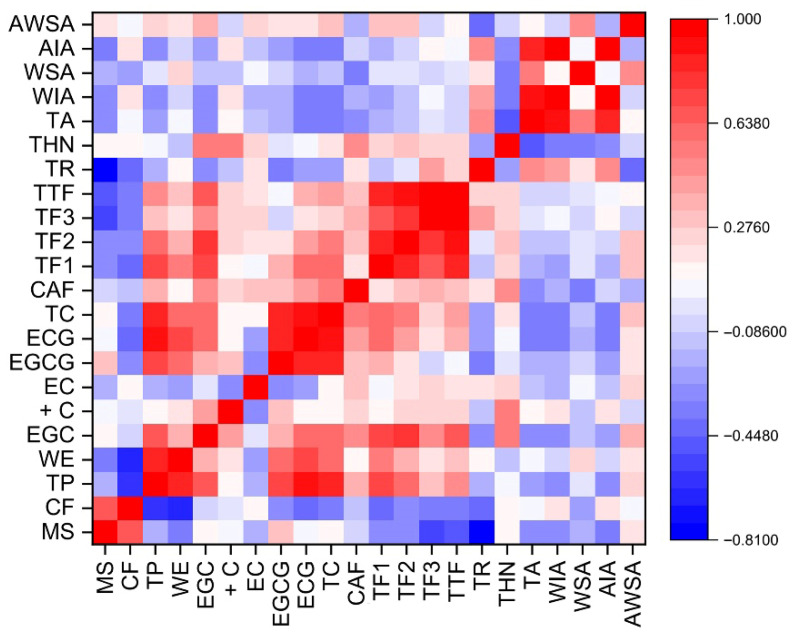
Pearson Correlation heatmaps between different biochemical parameters and the grade (sieve diameter) of orthodox black tea. MS, mesh size; CF, crude fibre; TP, total polyphenol; WE, water extract; EGC, (−)-epigallocatechin; +C, (+)-catechin; EC, (−)-epicatechin; EGCG, (−)-epigallocatechin-3-gallate; ECG, (−)-epicatechin-3-gallate; TC, total catechin; CAF, caffeine; TF1, theaflavin; TF2, theaflavin monogallate; TF3, theaflavin digallate; TTF, total theaflavin; TR, thearubigin; THN, theanine; TA, total ash; WIA, water insoluble ash; WSA, water soluble ash; AIA, acid insoluble ash; and AWSA, alkalinity of water soluble ash.

Pearson correlation analyses were performed to assess the relationships among the biochemical constituents of orthodox black tea, including the influence of sieve mesh size on chemical composition (Figure 5). Mesh size exhibited a significant positive correlation with CF content (R^2^ = 0.65, *p* ≤ 0.05), but significant negative correlations with TR, TF, TF3, and TP (all R^2^ = −0.65, *p* ≤ 0.05). CF showed strong negative correlations with TP (R^2^ = −0.58, *p* ≤ 0.05), WE (R^2^ = −0.65, *p* ≤ 0.05), TF (R^2^ = −0.37, *p* ≤ 0.05), and TR (R^2^ = −0.40, *p* ≤ 0.05). These patterns indicate that larger mesh sizes allow coarser shoots with higher fiber and lower polyphenolic content to pass through, resulting in reduced water extract and lower polyphenolic components compared with finer grades. TP demonstrated significant positive correlations with residual catechins (R^2^ = 0.74–0.91, *p* ≤ 0.05), reflecting the comparatively higher levels of intact catechins typically retained in orthodox teas. The ash profile showed weak to moderate negative correlations with TP (R^2^ = −0.26 – −0.30, *p* ≤ 0.05) and theanine (R^2^ = −0.27 – −0.46, *p* ≤ 0.05), but a significant positive correlation with TR (R^2^ = 0.44–0.51, *p* ≤ 0.05).

**Figure 6:**
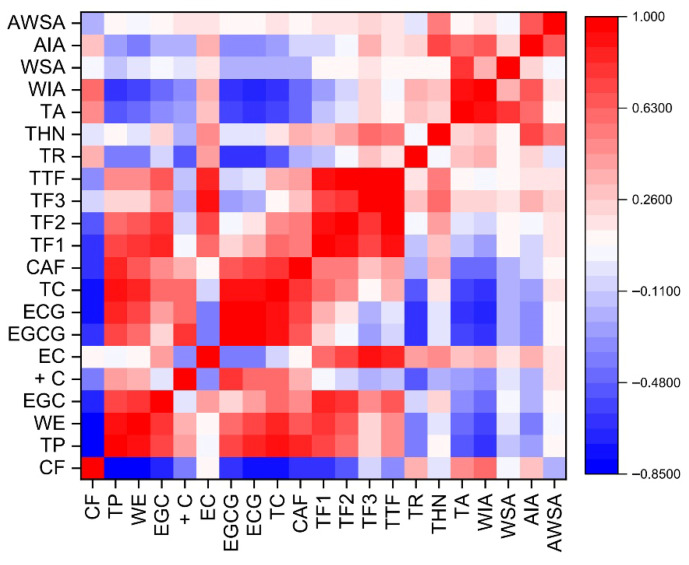
Pearson Correlation heatmaps between different biochemical parameters of CTC black tea. CF, crude fibre; TP, total polyphenol; WE, water extract; EGC, (−)-epigallocatechin; +C, (+)-catechin; EC, (−)-epicatechin; EGCG, (−)-epigallocatechin-3-gallate; ECG, (−)-epicatechin-3-gallate; TC, total catechin; CAF, caffeine; TF1, theaflavin; TF2, theaflavin monogallate; TF3, theaflavin digallate; TTF, total theaflavin; TR, thearubigin; THN, theanine; TA, total ash; WIA, water insoluble ash; WSA, water soluble ash; AIA, acid insoluble ash; and AWSA, alkalinity of water soluble ash.

In CTC teas, TP content showed significant positive correlations with WE, TC, caffeine, and TF, with correlation coefficients ranging from 0.48 to 0.92 (*p* ≤ 0.05) (Figure 6). Conversely, TP was strongly and negatively correlated with CF (R^2^ = −0.84, *p* ≤ 0.05) and total ash (R^2^ = −0.50, *p* ≤ 0.05). CF displayed significant negative correlations with WE, TC, and TF (R^2^ = −0.33 – −0.84), but positive correlations with TR, TA, and water-insoluble ash (R^2^ = 0.36–0.62). WE correlated positively with TF (R^2^ = 0.48) and negatively with TA (R^2^ = −0.42) and water-insoluble ash (R^2^ = −0.58). Theanine exhibited positive correlations with individual TF fractions and with TF (R^2^ = 0.31–0.58, *p* ≤ 0.05). Bhuyan et al. observed a similar level of positive correlation between TF and caffeine content in CTC black tea [9].

## 4. Conclusions

This study presents a picture of the black tea quality being produced in the two important regions of Assam in India. The TP, WE, CF, and ash characteristics of all samples satisfied the quality criteria specified by ISO and FSSAI, indicating overall conformity with established regulatory standards. Considering the dataset as a whole, the teas collectively exhibited desirable levels of TP, TF, WE, and caffeine, alongside CF values consistent with appropriate leaf maturity and processing practices. TR and theanine contents remained within a relatively narrow range across samples, suggesting stability of these key bioactive constituents under diverse cultivation and manufacturing conditions. Ash-related parameters, including water-soluble ash, acid-insoluble ash, and the alkalinity of water-soluble ash, were within ISO-accepted limits for all samples, further corroborating adherence to recognized quality norms. This investigation establishes that contemporary Assam black teas represent biochemically consistent, regulatory-compliant products of considerable market potential. Beyond confirming compliance, the data reveal substantial intra- and inter-regional variation reflecting agroclimatic and processing influences. Future research on agroclimatic-bioactive linkages with sensory and processing variabilities will strengthen the scientific foundation supporting both regulatory compliance and consumer health, which is essential for sustainable growth in the industry.

## Figures and Tables

**Table 1:** (**a**): Total polyphenol, water extract, caffeine, crude fibre, thearubigin, and theanine content of black tea samples of Upper Assam region. (**b**): Total polyphenol, water extract, caffeine, crude fibre, thearubigin, and theanine content of black tea samples of North Bank region.

(**a**)							
**Sample**	**Types**	**TP (mg g^−1^)**	**WE (mg g^−1^)**	**Caffeine (mg g^−1^)**	**CF (mg g^−1^)**	**TR (mg g^−1^)**	**Theanine (mg g^−1^)**
U1	Orthodox	107.74 ± 3.27 ^ij^	399.14 ± 2.23 ^d^	25.18 ± 1.89 ^fg^	119.54 ± 1.08 ^de^	139.67 ± 5.72 ^abc^	5.46 ± 0.17 ^cd^
U2	Orthodox	100.29 ± 0.28 ^jk^	373.02 ± 1.25 ^fg^	24.73 ± 3.44 ^fg^	118.16 ± 8.88 ^def^	115.10 ± 0.43 ^efg^	5.60 ± 0.04 ^c^
U3	CTC	97.39 ± 1.18 ^k^	380.97 ± 4.68 ^ef^	21.73 ± 6.22 ^gh^	150.16 ± 6.47 ^bc^	114.98 ± 3.30 ^efg^	3.99 ± 0.12 ^e^
U4	CTC	83.54 ± 1.68 ^l^	360.57 ± 4.57 ^h^	16.82 ± 1.32 ^i^	162.71 ± 2.08 ^a^	121.09 ± 11.64 ^def^	2.47 ± 0.08 ^h^
U5	Orthodox	143.22 ± 7.14 ^cd^	410.85 ± 6.74 ^c^	15.51 ± 1.07 ^i^	106.75 ± 4.75 ^ghi^	90.08 ± 7.05 ^hi^	3.91 ± 0.31 ^e^
U6	Orthodox	143.47 ± 3.27 ^cd^	423.81 ± 4.20 ^b^	17.98 ± 1.63 ^hi^	102.25 ± 0.41 ^hi^	101.06 ± 13.02 ^gh^	3.59 ± 0.22 ^ef^
U7	Orthodox	136.57 ± 5.31 ^de^	402.78 ± 1.71 ^cd^	30.60 ± 1.46 ^bcd^	116.96 ± 3.97 ^defg^	97.76 ± 6.15 ^gh^	2.76 ± 0.16 ^gh^
U8	Orthodox	148.56 ± 0.66 ^c^	431.29 ± 4.37 ^b^	33.54 ± 1.30 ^ab^	111.05 ± 2.26 ^efgh^	140.92 ± 16.25 ^ab^	2.86 ± 0.01 ^gh^
U9	Orthodox	120.50 ± 0.74 ^fgh^	380.25 ± 6.27 ^ef^	33.09 ± 0.39 ^abc^	124.28 ± 2.34 ^d^	123.40 ± 5.25 ^bcdef^	7.06 ± 0.04 ^b^
U10	Orthodox	121.10 ± 2.13 ^fg^	367.78 ± 2.61 ^gh^	33.14 ± 0.10 ^abc^	157.50 ± 7.33 ^ab^	73.95 ± 12.14 ^i^	7.27 ± 0.18 ^b^
U11	Orthodox	115.48 ± 0.95 ^ghi^	373.68 ± 3.16 ^fg^	33.41 ± 0.33 ^ab^	114.46 ± 4.67 ^defg^	111.22 ± 7.56 ^fg^	7.00 ± 0.15 ^b^
U12	Orthodox	128.17 ± 1.94 ^ef^	399.17 ± 6.25 ^d^	36.21 ± 0.32 ^a^	121.30 ± 7.64 ^de^	136.35 ± 13.62 ^abcd^	5.69 ± 0.05 ^c^
U13	Orthodox	166.46 ± 4.56 ^b^	445.80 ± 1.56 ^a^	35.57 ± 0.57 ^a^	97.33 ± 1.90 ^i^	122.21 ± 12.89 ^cdef^	7.18 ± 0.71 ^b^
U14	Orthodox	184.52 ± 3.91 ^a^	430.77 ± 3.34 ^b^	36.88 ± 0.41 ^a^	101.20 ± 1.78 ^hi^	90.73 ± 5.60 ^hi^	7.31 ± 0.44 ^b^
U15	CTC	108.57 ± 9.49 ^ij^	375.05 ± 4.41 ^efg^	28.17 ± 1.93 ^def^	157.21 ± 4.22 ^ab^	145.37 ± 6.22 ^a^	3.18 ± 0.08 ^fg^
U16	CTC	102.93 ± 6.08 ^jk^	376.36 ± 2.91 ^efg^	25.80 ± 1.12 ^efg^	145.99 ± 1.54 ^c^	141.74 ± 6.62 ^a^	7.23 ± 0.43 ^b^
U17	Orthodox	111.99 ± 2.92 ^hi^	380.09 ± 4.80 ^ef^	32.98 ± 1.99 ^abc^	107.59 ± 9.74 ^fghi^	140.11 ± 7.62 ^abc^	5.00 ± 0.17 ^d^
U18	Orthodox	108.03 ± 4.76 ^ij^	349.29 ± 9.07 ^i^	28.90 ± 0.99 ^cdef^	150.67 ± 4.76 ^bc^	86.09 ± 2.43 ^hi^	5.24 ± 0.14 ^cd^
U19	CTC	118.24 ± 2.59 ^gh^	383.99 ± 2.65 ^e^	27.98 ± 0.18 ^def^	155.15 ± 6.50 ^abc^	112.68 ± 2.66 ^fg^	8.16 ± 0.03 ^a^
U20	CTC	115.28 ± 1.25 ^ghi^	384.06 ± 1.73 ^e^	29.80 ± 0.14 ^bcde^	154.77 ± 3.93 ^abc^	132.29 ± 5.08 ^abcde^	8.07 ± 0.09 ^a^
(**b**)							
**Sample**	**Types**	**TP (mg g^−1^)**	**WE (mg g^−1^)**	**Caffeine (mg g^−1^)**	**CF (mg g^−1^)**	**TR (mg g^−1^)**	**Theanine (mg g^−1^)**
N1	Orthodox	152.91 ± 10.64 ^cd^	406.58 ± 8.61 ^c^	37.40 ± 1.89 ^ab^	91.87 ± 3.84 ^e^	116.44 ± 3.13 ^bc^	6.69 ± 0.06 ^b^
N2	Orthodox	148.97 ± 1.28 ^cd^	411.71 ± 1.83 ^bc^	35.74 ± 1.28 ^bc^	93.95 ± 2.43 ^e^	115.70 ± 4.65 ^bc^	5.61 ± 0.02 ^e^
N3	Orthodox	139.28 ± 3.23 ^de^	406.76 ± 3.69 ^c^	30.75 ± 0.50 ^efgh^	127.07 ± 5.00 ^bc^	59.62 ± 4.73 ^f^	6.22 ± 0.19 ^c^
N4	Orthodox	148.09 ± 2.56 ^cd^	431.08 ± 4.73 ^a^	32.94 ± 1.13 ^cdef^	118.88 ± 3.10 ^c^	87.09 ± 5.64 ^e^	7.49 ± 0.07 ^a^
N5	Orthodox	131.67 ± 4.84 ^e^	428.92 ± 13.33 ^ab^	31.43 ± 1.19 ^defg^	118.69 ± 2.14 ^c^	127.91 ± 7.01 ^ab^	5.43 ± 0.07 ^e^
N6	CTC	151.04 ± 9.48 ^cd^	440.50 ± 7.49 ^a^	29.86 ± 1.42 ^fgh^	139.19 ± 4.49 ^a^	118.23 ± 10.27 ^bc^	6.09 ± 0.06 ^cd^
N7	CTC	140.77 ± 2.79 ^de^	434.64 ± 5.36 ^a^	28.10 ± 0.54 ^h^	140.77 ± 7.55 ^c^	137.84 ± 11.86 ^a^	6.01 ± 0.11 ^d^
N8	CTC	150.66 ± 7.49 ^cd^	442.51 ± 12.36 ^a^	28.68 ± 1.74 ^gh^	130.80 ± 2.12 ^ab^	127.19 ± 10.31 ^ab^	3.61 ± 0.04 ^h^
N9	Orthodox	180.85 ± 7.15 ^a^	434.03 ± 10.92 ^a^	39.24 ± 1.49 ^a^	104.76 ± 2.95 ^d^	113.61 ± 1.66 ^bc^	5.53 ± 0.15 ^e^
N10	CTC	154.01 ± 8.21 ^cd^	436.77 ± 12.25 ^a^	33.23 ± 0.92 ^cde^	129.51 ± 7.13 ^ab^	119.50 ± 3.16 ^bc^	5.17 ± 0.08 ^f^
N11	CTC	160.23 ± 9.18 ^bc^	432.02 ± 14.87 ^a^	33.26 ± 2.33 ^cde^	117.63 ± 1.31 ^c^	108.09 ± 4.29 ^cd^	6.08 ± 0.04 ^cd^
N12	CTC	169.74 ± 3.10 ^ab^	447.54 ± 9.85 ^a^	34.29 ± 0.66 ^bcd^	117.39 ± 3.61 ^c^	95.71 ± 3.40 ^de^	4.09 ± 0.09 ^g^

TP, total polyphenol; WE, water extract; CF, crude fibre; TR, thearubigin. Values are ‘mean ± standard deviation’ of independent triplicate measurements. The same superscript letters within a column denotes not significant, whereas a different letter denotes a significant difference. Means were compared using Tukey’s multiple comparison tests at *p* ≤ 0.05.

**Table 2:** (**a**): Ash profile of black tea samples from Upper Assam region. (**b**): Ash profile of black tea samples from North Bank region.

(**a**)						
**Sample**	**Types**	**Water Insoluble (%)**	**Water Soluble (%)**	**Total (%)**	**Acid Insoluble (%)**	**Alkalinity of Water Soluble Ash (g KOH Equivalent)**
U1	Orthodox	3.97 ± 0.31 ^a^	4.45 ± 0.15 ^ab^	8.42 ± 0.45 ^a^	1.98 ± 0.35 ^a^	2.05 ± 0.05 ^def^
U2	Orthodox	2.62 ± 0.87 ^b^	3.87 ± 0.78 ^b^	6.49 ± 0.16 ^cdef^	0.26 ± 0.09 ^def^	2.02 ± 0.03 ^efg^
U3	CTC	2.31 ± 0.13 ^bcd^	4.21 ± 0.17 ^ab^	6.52 ± 0.30 ^cdef^	0.05 ± 0.04 ^f^	2.00 ± 0.21 ^efg^
U4	CTC	2.21 ± 0.07 ^bcde^	4.00 ± 0.03 ^ab^	6.21 ± 0.04 ^cdefg^	0.19 ± 0.03 ^ef^	2.13 ± 0.03 ^bcdef^
U5	Orthodox	2.08 ± 0.25 ^cde^	4.43 ± 0.54 ^ab^	6.51 ± 0.76 ^cdef^	0.18 ± 0.03 ^f^	2.19 ± 0.24 ^abcde^
U6	Orthodox	2.34 ± 0.03 ^bcd^	4.33 ± 0.06 ^ab^	6.67 ± 0.05 ^cd^	0.24 ± 0.03 ^def^	2.32 ± 0.01 ^ab^
U7	Orthodox	2.22 ± 0.06 ^bcde^	4.55 ± 0.20 ^a^	6.76 ± 0.23 ^c^	0.05 ± 0.01 ^f^	2.35 ± 0.03 ^ab^
U8	Orthodox	3.83 ± 0.28 ^a^	4.23 ± 0.04 ^ab^	8.06 ± 0.32 ^ab^	1.50 ± 0.25 ^b^	2.16 ± 0.03 ^abcde^
U9	Orthodox	2.06 ± 0.24 ^cde^	4.52 ± 0.59 ^a^	6.58 ± 0.83 ^cde^	0.15 ± 0.02 ^f^	2.32 ± 0.30 ^ab^
U10	Orthodox	2.05 ± 0.11 ^cde^	4.02 ± 0.13 ^ab^	6.08 ± 0.05 ^defgh^	0.19 ± 0.11 ^ef^	2.34 ± 0.08 ^ab^
U11	Orthodox	1.97 ± 0.09 ^cde^	3.96 ± 0.05 ^ab^	5.93 ± 0.05 ^efgh^	0.19 ± 0.04 ^ef^	2.14 ± 0.01 ^bcdef^
U12	Orthodox	3.63 ± 0.35 ^a^	3.89 ± 0.07 ^b^	7.52 ± 0.31 ^b^	1.89 ± 0.34 ^a^	2.04 ± 0.02 ^def^
U13	Orthodox	1.91 ± 0.02 ^de^	4.13 ± 0.07 ^ab^	6.04 ± 0.07 ^defgh^	0.14 ± 0.04 ^f^	2.08 ± 0.01 ^cdef^
U14	Orthodox	1.83 ± 0.04 ^de^	3.99 ± 0.04 ^ab^	5.82 ± 0.07 ^gh^	0.04 ± 0.01 ^f^	2.37 ± 0.08 ^a^
U15	CTC	2.07 ± 0.02 ^cde^	3.84 ± 0.05 ^b^	5.91 ± 0.07 ^fgh^	0.02 ± 0.01 ^f^	1.92 ± 0.06 ^fg^
U16	CTC	2.32 ± 0.07 ^bcd^	4.13 ± 0.01 ^ab^	6.45 ± 0.05 ^cdefg^	0.46 ± 0.02 ^cde^	2.26 ± 0.09 ^abcd^
U17	Orthodox	1.78 ± 0.01 ^e^	4.31 ± 0.10 ^ab^	6.08 ± 0.10 ^defgh^	0.15 ± 0.01 ^f^	1.80 ± 0.02 ^g^
U18	Orthodox	2.32 ± 0.07 ^bcd^	3.20 ± 0.28 ^c^	5.53 ± 0.21 ^h^	0.68 ± 0.10 ^c^	1.91 ± 0.03 ^fg^
U19	CTC	2.49 ± 0.02 ^bc^	3.97 ± 0.18 ^ab^	6.46 ± 0.19 ^cdefg^	0.51 ± 0.03 ^cd^	2.21 ± 0.05 ^abcde^
U20	CTC	2.35 ± 0.03 ^bcd^	4.08 ± 0.04 ^ab^	6.43 ± 0.06 ^cdefg^	0.51 ± 0.04 ^cd^	2.28 ± 0.04 ^abc^
**(b)**						
**Sample**	**Types**	**Water Insoluble (%)**	**Water Soluble (%)**	**Total (%)**	**Acid Insoluble (%)**	**Alkalinity of Water Soluble Ash (g KOH Equivalent)**
N1	Orthodox	1.81 ± 0.12 ^de^	3.78 ± 0.27	5.59 ± 0.21 ^e^	0.10 ± 0.01 ^def^	1.89 ± 0.05 ^f^
N2	Orthodox	1.89 ± 0.02 ^cde^	3.87 ± 0.11	5.76 ± 0.11 ^cde^	0.12 ± 0.03 ^cde^	2.15 ± 0.03 ^abc^
N3	Orthodox	1.98 ± 0.13 ^bcd^	4.18 ± 0.17	6.16 ± 0.23 ^ab^	0.04 ± 0.01 ^f^	2.17 ± 0.07 ^ab^
N4	Orthodox	2.13 ± 0.21 ^ab^	3.84 ± 0.10	5.97 ± 0.11 ^abcd^	0.04 ± 0.01 ^f^	2.03 ± 0.05 ^e^
N5	Orthodox	1.70 ± 0.06 ^e^	3.96 ± 0.01	5.66 ± 0.06 ^de^	0.05 ± 0.01 ^f^	2.02 ± 0.04 ^e^
N6	CTC	2.03 ± 0.10 ^bc^	4.03 ± 0.33	6.05 ± 0.31 ^abc^	0.05 ± 0.01 ^ef^	2.03 ± 0.04 ^de^
N7	CTC	2.13 ± 0.03 ^ab^	4.12 ± 0.15	6.25 ± 0.17 ^ab^	0.23 ± 0.03 ^a^	2.25 ± 0.03 ^a^
N8	CTC	2.25 ± 0.02 ^a^	4.09 ± 0.14	6.34 ± 0.14 ^a^	0.18 ± 0.03 ^abc^	2.13 ± 0.07 ^bcd^
N9	Orthodox	1.88 ± 0.06 ^cde^	3.88 ± 0.16	5.76 ± 0.13 ^cde^	0.20 ± 0.03 ^ab^	2.06 ± 0.04 ^cde^
N10	CTC	2.08 ± 0.09 ^abc^	3.95 ± 0.09	6.03 ± 0.18 ^abc^	0.15 ± 0.03 ^bcd^	2.11 ± 0.05 ^bcde^
N11	CTC	1.72 ± 0.02 ^e^	3.89 ± 0.01	5.62 ± 0.03 ^de^	0.10 ± 0.07 ^def^	2.13 ± 0.03 ^bcd^
N12	CTC	1.91 ± 0.04 ^cde^	4.00 ± 0.01	5.91 ± 0.05 ^bcde^	0.10 ± 0.02 ^def^	2.20 ± 0.01 ^ab^

Values are ‘mean ± standard deviation’ of independent triplicate measurements. The same superscript letters within a column denotes not significant, whereas a different letter denotes a significant difference. Means were compared using Tukey’s multiple comparison tests at *p* ≤ 0.05.

## Data Availability

The original contributions presented in this study are included in the article/Supplementary Materials. Further inquiries can be directed to the corresponding authors.

## Supplementary Materials

The following supporting information can be downloaded at: https://www.mdpi.com/article/10.3390/foods15010158/s1, Table S1: Weather Data of the sample collected region. Table S2: Catechin profile of black tea samples from the upper Assam region. Table S3: Catechin profile of black tea samples from the North bank region. Table S4: Black tea sample code and their corresponding types of tea and grade.
