# Supplementary material for: Biochemical Quality Profile of Black Tea from Upper Assam and North Bank Region of Assam, India
Source: Foods. 2026 Jan 3;15(1):158. doi: 10.3390/foods15010158 (PMC12785656; doi:10.3390/foods15010158)
Supplement: Supplementary file 1 [file foods-15-00158-s001.zip › foods-4051008-supplementary.pdf]

Supplementary Information

Biochemical Quality Profiles of Black Teas from Upper Assam and the North Bank Regions of Assam, India

Podma Pollov Sarmah[a,1,*], Himangshu Deka[a,1,*], Priyanuj Parasar[1], Rashmi Baruah[2], Santanu Sabhapondit[1], Dibyajit Buragohain[3]

[1]Biochemistry Department, Tocklai Tea Research Institute, Jorhat, Assam 785008, India

[2]College of Sericulture, Assam Agricultural University, Jorhat, Assam 785013. India

[3]Advisory Department, Tocklai Tea Research Institute, Jorhat, Assam 785008, India

[a] These two authors contributed equally to this work.

*Correspondence: p.pollov@tocklai.net; podmapollov@gmail.com (PP Sarmah)
deka.himangshu@tocklai.net; himangshu1234@gmail.com (H Deka)

Table S1: Weather Data of the sample collected region

Region	Month	Maximum Temperature (°C)	Minimum Temperature (°C)	Total Rainfall (cm)	Relative Humidity (%)		Sunshine hours
					Morning	Afternoon	
North Bank	April	30.7	19.8	84.9	87.0	56.0	5.9
	May	31.0	21.6	125.4	87.0	60.0	6.2
Upper Assam	April	28.7	19.3	157.6	90	62	4.5
	May	28.8	21.1	208.9	89	69	4.7

Table S2 : Catechin profile of black tea samples from the upper Assam region

Sample	Types	EGC (mg g^{-1})	+ C (mg g^{-1})	EC (mg g^{-1})	EGCG (mg g^{-1})	ECG (mg g^{-1})	TC (mg g^{-1})
U1	Orthodox	ND	0.33 ±0.03	0.00	1.02 ±0.28	1.12 ±0.17	2.48 ±0.24
U2	Orthodox	ND	0.17 ±0.12	4.16 ±0.73	0.44 ±0.16	1.26 ±0.21	6.03 ±1.21
U3	CTC	ND	ND	0.00	0.47 ±0.09	0.54 ±0.26	1.00 ±0.32
U4	CTC	ND	ND	ND	ND	ND	ND
U5	Orthodox	ND	ND	1.83 ±0.08	3.15 ±0.38	5.14 ±0.47	10.51 ±0.91
U6	Orthodox	ND	ND	3.12 ±0.99	3.17 ±1.20	4.63 ±0.58	10.91 ±2.06
U7	Orthodox	ND	ND	3.86 ±0.69	6.51 ±0.22	8.94 ±0.29	19.32 ±1.19
U8	Orthodox	ND	ND	4.40 ±0.04	4.40 ±0.09	8.23 ±0.36	17.02 ±0.43
U9	Orthodox	0.48 ±0.31	0.18 ±0.03	7.40 ±0.66	1.53 ±.21	2.32 ±0.11	11.92 ±1.00
U10	Orthodox	0.91 ±0.29	ND	7.18 ±0.06	2.16 ±0.06	3.28 ±0.01	13.53 ±0.26
U11	Orthodox	ND	0.21 ±0.08	6.08 ±0.33	1.48 ±0.40	1.74 ±0.25	9.52 ±0.19
U12	Orthodox	0.37 ±0.08	0.34 ±0.09	6.80 ±0.11	2.58 ±0.07	2.11 ±0.20	12.20 ±0.18
U13	Orthodox	0.84 ±0.14	0.11 ±0.03	5.45 ±0.21	4.28 ±0.10	11.60 ±0.56	22.28 ±0.66
U14	Orthodox	1.94 ±0.05	0.33 ±0.03	5.81 ±0.52	5.41 ±0.09	13.07 ±0.16	26.55 ±0.30
U15	CTC	ND	ND	2.96 ±0.27	1.27 ±0.04	ND	4.23 ±0.31
U16	CTC	0.42 ±0.18	ND	3.71 ±0.20	1.75 ±0.11	0.97 ±0.09	6.85 ±0.52
U17	Orthodox	ND	ND	5.07 ±0.51	0.55 ±0.11	0.61 ±0.06	6.23 ±0.68
U18	Orthodox	ND	ND	2.81 ±0.21	2.42 ±0.09	4.49 ±0.12	9.72 ±0.39
U19	CTC	0.36 ±0.09	ND	4.44 ±0.20	1.96 ±0.08	2.35 ±0.06	9.10 ±0.43
U20	CTC	0.38 ±0.01	ND	3.81 ±0.05	0.69 ±0.08	0.39 ±0.05	5.28 ±0.04

*EGC, (−)-epigallocatechin; +C, (+)-catechin; EC, (−)-epicatechin; EGCG, (−)-epigallocatechingallate; ECG, (−)-epicatechingallate; TC, total catechin; ND, not detected. Values are 'mean±standard deviation' of independent triplicate measurements.

Table S3: Catechin profile of black tea samples from the North bank region

Sample	Types	EGC (mg g^{-1})	+ C (mg g^{-1})	EC (mg g^{-1})	EGCG (mg g^{-1})	ECG (mg g^{-1})	TC (mg g^{-1})
N1	Orthodox	ND	0.11 ±0.02	4.61 ±0.57	6.33 ±0.13	11.64 ±0.50	22.69 ±1.19
N2	Orthodox	0.17 ±0.06	ND	3.13 ±0.26	2.26 ±0.07	6.57 ±0.30	12.12 ±0.66
N3	Orthodox	0.58 ±0.25	0.25 ±0.03	2.31 ±0.11	4.08 ±0.07	8.07 ±0.26	15.30 ±0.52
N4	Orthodox	0.69 ±0.06	0.67 ±0.04	ND	7.06 ±0.29	8.21 ±0.40	16.63 ±0.66
N5	Orthodox	ND	ND	6.27 ±0.75	1.80 ±0.11	5.50 ±0.16	13.58 ±1.03
N6	CTC	2.90 ±0.25	ND	6.67 ±0.50	3.26 ±0.20	5.02 ±0.27	17.85 ±1.16
N7	CTC	2.98 ±0.19	ND	5.00 ±0.10	1.43 ±0.08	2.74 ±0.13	12.16 ±0.38
N8	CTC	1.32 ±0.33	ND	4.08 ±0.35	0.86 ±0.10	2.10 ±0.13	8.35 ±0.90
N9	Orthodox	1.46 ±0.17	0.23 ±0.03	ND	5.20 ±0.16	13.35 ±0.38	20.24 ±0.67
N10	CTC	1.39 ±0.13	ND	ND	6.45 ±0.26	10.02 ±0.43	17.86 ±0.81
N11	CTC	1.93 ±0.33	ND	ND	6.44 ±0.55	10.37 ±0.89	18.75 ±1.76
N12	CTC	0.89 ±0.20	0.09 ±0.03	ND	12.34 ±0.39	13.01 ±0.35	26.33 ±0.75

*EGC, (−)-epigallocatechin; +C, (+)-catechin; EC, (−)-epicatechin; EGCG, (−)-epigallocatechingallate; ECG, (−)-epicatechingallate; TC, total catechin; ; ND, not detected. Values are 'mean±standard deviation' of independent triplicate measurements.

Table S4: Black tea sample code and their corresponding types of tea and grade.

Upper Assam Region				North Bank Region			
**Sample Code**	**Type**	**Grade**	**GPS coordinate of sample collection site**	**Sample Code**	**Type**	**Grade**	**GPS coordinate of sample collection site**
U-1	Orthodox	OD	27°38'38.2"N 95°30'06.7"E	N-1	Orthodox	GFBOP	27°21'33.8"N 94°06'08.1"E
U-2	Orthodox	GBOP	27°38'38.2"N 95°30'06.7"E	N-2	Orthodox	OPD	27°21'33.8"N 94°06'08.1"E
U-3	CTC	BPSM	27°30'4.60"N 95°0'46.05"E	N-3	Orthodox	TGFOP	27°21'33.8"N 94°06'08.1"E
U-4	CTC	PD	27°30'4.60"N 95°0'46.05"E	N-4	Orthodox	TGFOP1	27°21'33.8"N 94°06'08.1"E
U-5	Orthodox	GFBOP	27°08'07.8"N 94°49'06.4"E	N-5	Orthodox	BOPF	27°21'33.8"N 94°06'08.1"E
U-6	Orthodox	GOF	27°08'07.8"N 94°49'06.4"E	N-6	CTC	BOP(SM)	26°50'41.4"N 92°37'49.6"E
U-7	Orthodox	TGFOP	27°08'07.8"N 94°49'06.4"E	N-7	CTC	PF	26°50'41.4"N 92°37'49.6"E
U-8	Orthodox	OD	27°08'07.8"N 94°49'06.4"E	N-8	CTC	PD	26°50'41.4"N 92°37'49.6"E
U-9	Orthodox	GBOP	27°29'35.0"N 95°15'23.2"E	N-9	Orthodox	GOF	26°46'15.6"N 93°16'26.4"E
U-10	Orthodox	TGFOP	27°29'35.0"N 95°15'23.2"E	N-10	CTC	PF	26°52'07.7"N 92°41'30.3"E
U-11	Orthodox	BOPF	27°29'35.0"N 95°15'23.2"E	N-11	CTC	PD	26°52'07.7"N 92°41'30.3"E
U-12	Orthodox	OD	27°29'35.0"N 95°15'23.2"E	N-12	CTC	BOP(SM)	26°52'07.7"N 92°41'30.3"E
U-13	Orthodox	OPD	26°46'56.7"N 93°16'04.6"E				
U-14	Orthodox	GFBOP	26°46'56.7"N 93°16'04.6"E				
U-15	CTC	PF	27°30'4.60"N 95°0'46.05"E				
U-16	CTC	PD	27°28'22.7"N 94°54'53.1"E				
U-17	Orthodox	BOPF	27°38'38.2"N 95°30'06.7"E				
U-18	Orthodox	TGFOP	27°38'38.2"N 95°30'06.7"E				
U-19	CTC	BPSM	27°28'22.7"N 94°54'53.1"E				
U-20	CTC	PF	27°28'22.7"N 94°54'53.1"E				
